# Molecular identification and phylogenetic analysis of confusing *Tetrastigma* species based on DNA barcoding and chloroplast genome

**DOI:** 10.3389/fphar.2025.1607947

**Published:** 2025-07-11

**Authors:** Xianjing Li, Yue Zhang, Meifang Song, Niaojiao Xu, Lu Qu, Haitao Li, Yunqiang Wang, Baozhong Duan, Zhonglian Zhang

**Affiliations:** ^1^ College of Pharmacy, Dali University, Dali, China; ^2^ Yunnan Key Laboratory of Southern Medicine Utilization, Yunnan Branch of Institute of Medicinal Plant Development Chinese Academy of Medical Sciences, Peking Union Medical College, Jinghong, China

**Keywords:** *Tetrastigma*, *Tetrastigma obtectum*, *Tetrastigma serrulatum*, chloroplast genome, species identification, phylogenetic relationship

## Abstract

**Background:**

*Tetrastigma* plants are widely utilized in traditional medicine (such as *Tetrastigma. obtectum* and *Tetrastigma. serrulatum,* two important commonly medicinal plants), primarily for their properties in promoting blood circulation, strengthening bones and tendons, and so on. However, the high diversity of species differentiation poses a challenge in accurately identifying the various *Tetrastigma* species without specialized taxonomic knowledge.

**Materials and methods:**

To screen the candidate barcode sequences of *Tetrastigma* species, we first report the complete chloroplasts (CP) genomes of *T*. *obtectum* and *T*. *serrulatum* obtained via high throughput Illumina sequencing and compare them with fourteen previously sequenced species. Furthermore, we collected fresh leaf samples from *T. obtectum* and *T. serrulatum* (totally 37 samples) and evaluated the discriminatory efficacy of the nuclear DNA Internal Transcribed Spacer 2 (ITS2) fragment through comparative analysis of sequence variations and secondary structures. Finally, to analyze the phylogenetic position of *Tetrastigma* species, we constructed a Maximum Likelihood (ML) phylogenetic tree using CP genome sequences of 46 species from seven genera within the Vitaceae family.

**Results and discussion:**

The CP genomes of *Tetrastigma* exhibited a typical circular tetramerous structure, including a large single-copy region (LSC) (87,381–88,979 bp), a small single-copy region (SSC) (18,649–19,339 bp), and a pair of inverted repeats (IRa and IRb) (26,288–26,934 bp). The guanine-cytosine content of the CP genomes is 37.35%–37.62%. The codon usage shows a significant preference for end with A/T. Then, the results of nucleotide diversity analysis showed that ten polymorphic hotspots (*psb*M-*trn*D-GUC, *ndh*F-*rpl*32, *trn*S-GCU-*trn*G-UCC, *ycf*1, *rpl*32-*trn*L-UAG, *trn*S-UGA-*psb*Z, *psb*E-*pet*L, *mat*K-*rps*16, *rpl*16, and *rpl*22) could be the candidate DNA marker suitable for *Tetrastigma* species. Furthermore, our results demonstrate that the ITS2 sequence could effectively discriminate *T. obtectum* and *T. serrulatum*, whereas the secondary structure cannot, proving that ITS2 can be used as an efficient barcode fragment to accurately identify the two species. The aim of this study was not only to determine the identification efficiency of the CP genome and ITS2 for *T. obtectum* and *T. serrulatum* but also to clarify the phylogenetic relationship and screen the candidate DNA marker suitable for *Tetrastigma* species, provide valuable data support for further accurate identification of the *Tetrastigma* genus.

## 1 Introduction

*Tetrastigma obtectum* (Wall.) Planch. Belongs to the genus *Tetrastigma* (Miq.) Planch. (Vitaceae), is an evergreen liana plant found mainly in China, Bhutan, Nepal, and Vietnam. It is a crucial commonly medicinal plant used in folk, which primarily had the functions of promoting blood circulation and clearing collaterals, reinforcing bones and tendons, clearing heat and cooling blood, etc., and had different applications in different nationalities (Table 1), such as Miao nationality, Naxi nationality, Tujia nationality, Yao nationality, and Yi nationality. However, due to the high diversity of species differentiation and the relatively concentrated distribution area of the species, it is difficult to accurately identify the genus without all the species knowledge in a relatively large area, and the phenomenon of invalid naming is very likely to occur (Li and Wu, 1995). The same is true for *T. obtectum*, especially for medicinal materials often found on the market. During our field investigation, we found that *T. obtectum* is usually confused with similar plant species of the same genus in the medicinal market, such as *Tetrastigma serrulatum* (Roxb.) Planch, residents often confuse the two species because of their similar appearance, similar distribution areas, similar habitat environment, and primary distribution in multi-ethnic regions. *T. serrulatum* could also be used as a medicinal material, and it appears to have a similar medicinal value to *T. obtectum*. Previous studies of our research group showed significant differences between *T. serrulatum* and *T. obtectum* in terms of compound composition, active ingredients, and pharmacological activities (The data has not yet been published). If the two are confused, it will inevitably affect the clinical application effect and have a non-negligible adverse impact on the patient’s health recovery and the further development of the medicinal material industry of the two species.

**TABLE 1:** Folk uses of *Tetrastigma obtectum* (Wall.) Planch.

User nations	Folk names	Medicinal parts	Folk use	References
Miao nationality	Yanwujia Yanpateng Zhuajianjinyong	Whole plant	Treat rheumatism pain, injury, swelling, sore poison, headache, lumbago, and limb pain	Wei et al. (1999), Zhang et al. (1998), Yang et al. (1998)
Naxi nationality	Xiaowuzhuajinlong	Root or Whole plant	Treat cervical lymph node tuberculosis, nameless swelling poison, tinea capitis, peripheral neuritis, rheumatic muscle and bone pain, rheumatic paralysis, headache, body pain, yellow water sore, sore poison, walking wind, fluid, fall injury, and fracture	Lin, (1991)
Tujia nationality	Wuzhuaqi Yanwujia Zouyoucao	Whole plant	Treatment of injury, irregular menstruation, cold disease, belt disease, osteomyelitis, rheumatism, joint swelling pain, tabes injury erysipelas, osteomyelitis, lumbago, herpes, snake bite, carbuncular ulcer	Yang et al. (2000), Yi et al. (1991), Song and Wan, (2003)
Yao nationality	Wuzhualong	Whole plant	Treatment of rheumatic joint pain, headache, and shingles	Ruan, (2002)
Yi nationality	Wumoliegu, muzhuteng, xiaohongnpao	Roots, stems, leaves, or Whole plant	Root: Treatment of fractures, joint dislocation; Stems, leaves: Treatment of broken bones, snake bites; Beaded root tuber: Treatment of fracture, knife wound hematoma, bruising injury, strain, tinea, madness Whole plant: treatment of fatigue, weak, madness, bruise injury, ringworm, fracture, knife wound hematoma	Hai et al. (1997), Bu, (1996), Wan, (1990), Xiao and Li, (1999)

As one of the important organelles in green plants, chloroplasts (CP) responsible for metabolic and photosynthetic processes, provide essential energy for plant growth and development (Daniell et al., 2016; Dobrogojski et al., 2020). Because of the relatively conserved genome structure characteristics that with a closed circular DNA molecule composed of a typical quadripartite structure: a large single-copy region (LSC), a small single-copy region (SSC), and a pair of mirrored inverted repeat sequences (IRa and IRb) (Sato et al., 1999; Ferrarini et al., 2013; Ahmad et al., 2022), CP genomes are often used for revealing evolutionary patterns and phylogenetic relationships of interspecies and intraspecies (Fan and Ma, 2022; Wicke et al., 2011; Jin and Daniell, 2015; Daniell et al., 2021). Researchers have demonstrated that the evolutionary rate of synonymous substitution in the CP genome is half that of the nuclear genome (Wang et al., 2024). Furthermore, the whole CP genome as a super-barcode has been widely used in species identification (Zhang et al., 2024; Chen et al., 2010), and the sequences selected from the highly-variation regions of the whole CP genome have been used for distinguishing closely related species and counterfeit medicinal materials, especially in tightly related taxa (Li et al., 2015; Yang et al., 2021; Zhang et al., 2024). In addition, the cognomen, characterized by maternal inheritance, is an ideal molecular data resource widely employed in research fields such as genetic engineering, breeding, and evolutionary biology (Wei et al., 2021; Dong et al., 2012). Therefore, investigating the CP genome is important for revealing evolutionary patterns, phylogenetic relationships, species identification, molecular breeding, and so on (Jin and Daniell, 2015; Daniell et al., 2021).

In addition, DNA barcoding has been applied more and more widely because of the overcoming of the limitations of traditional identification methods and allowed accurate identification of materials with similar morphologies and chemical structures (Gregory, 2005). In various DNA barcodes, the ribosomal DNA Internal Transcribed Spacer 2 (ITS2) sequence has been widely used to identify plant species because of its universality and specificity (Chen et al., 2023). Chen et al. (2010) first proposed the ITS2 region as an efficient barcoding tool for medicinal plants and their closely related species due to its relatively short length, consistent performance in distinguishing closely related species, and ease of amplification with a single set of universal primers, they also proposed that ITS2 can serve as a novel universal barcode for the identification of a broader range of plant taxa, they tested the discrimination ability of ITS2 in more than 6,600 plant samples belonging to 4,800 species from 753 distinct genera. They found that the rate of successful identification with the ITS2 was 92.7% at the species level. Yao et al. (2010) subsequently proposed ITS2 as a DNA barcode for plant and animal medicine based on an investigation of delimitation ability in 50,790 plants and 12,221 animals. The research results show that the success rates of the ITS2 region in identifying different taxonomic groups were 67.1%–91.7% at the species level (Yao et al., 2010). In sum, ITS2 remains the best and most widely used single-locus barcode for the delimitation and identification of medicinal plants, as it is easy to amplify and has enough variability to distinguish even closely related species (Han et al., 2016; Hu et al., 2022; Yao et al., 2022; Ma et al., 2017), despite the peculiarity of multiple-copy sequence and the identification ability differs among species and taxonomic levels (Sokołowska et al., 2022). Moreover, research on molecular markers aids in identifying the sources of adulterated medicinal materials, thus addressing market chaos and the challenges in tracing the origins of these materials. This has a significant positive impact on the safety of clinical medications and the recovery of patients’ health.

Here, we first sequenced the complete CP genomes of *T. obtectum* and its adulterant species *T. serrulatum*, using the Illumina HiSeq4000 sequencing platform. We assembled and annotated the CP genomes of the 2 *Tetrastigma* species. We compared them with 14 previously sequenced *Tetrastigma* species, examining their structural differences and identifying polymorphic hotspots suitable for candidate DNA marker development. Furthermore, we analyzed the published CP genomes of the Vitaceae family, constructing phylogenetic trees based on methods such as Maximum Likelihood (ML), and examined their phylogenetic relationships. Finally, we evaluated the identification efficiency of ITS2 fragments for 37 collected *T. obtectum and T. serrulatum* samples (17 and 20 samples, respectively). This study aimed to determine the identification efficiency of CP genome and DNA barcode fragment ITS2 for *T. obtectum* and *T. serrulatum*, and to clarify the relationship between the phylogenetic analysis.

## 2 Materials and methods

### 2.1 Plant material, DNA extraction and sequencing

*T. obtectum* and *T. serrulatum* collected in Yunnan. Fresh green leaves were sampled, washed, dried, and stored at −80°C till DNA extraction. The voucher specimens were deposited in the herbarium, Yunnan branch of the Institute of Medicinal Plant Development (IMPLAD), Chinese Academy of Medical Sciences herbarium (voucher numbers: IMDY2022091005, IMDY2022100011) and identified by Zhonglian Zhang. Total genomic DNA was extracted from fresh leaves using the TIANGEN plant Genomic DNA kit (Tianjin Biotech, Beijing, Co., Ltd.) The purity of total DNA was evaluated using electrophoresis on 1.0% agarose gels. And the concentration was measured using a Nanodrop spectrophotometer 2000 (Thermo Fisher Scientific Inc., Waltham, MA, United States). The OD260/280 value ranges from 1.8 to 2.2, and ≥2 µg was equally pooled from individuals that could be used to construct the library. The DNA was sheared into fragments at approximately 500 bp long using the Covaris M220 focused ultrasonicator (Covaris, Woburn, MA, United States) for paired-end library construction. The DNA library was constructed using the Illumina TruSeq™ Nano DNA Sample Prep Kit (Illumina, San Diego, CA, United States). The library was sequenced using the Illumina HiSeq4000 sequencing platform at Biozeron (Shanghai, China).

### 2.2 Genome assembly and annotations

The number of raw reads obtained for *T. obtectum* and *T. serrulatum* were checked (Q ≥ 25) using the Fast QC Toolkit (Brown et al., 2017). Low quality reads were filtered out from the raw data to obtain high-quality data (clean reads) for subsequent analysis, with the published CP genome of *Tetrastigma* species *T*. *hemsleyanum* and *T*. *planicaule* as the reference sequence. The CP genome was *De novo* assembled using Get Organelle (Jin et al., 2020), perform local assembly and optimization of the assembly results, as well as internal hole repairs. Producing circular assemblies for *T. obtectum* and *T. serrulatum* that conform to the quadripartite structure of CP genomes. The filtered files were visualized in Bandage v.0.8.1 (Wick et al., 2015). Bowite 2 in Geneious v.9.0.2 (Kearse et al., 2012) was used to align the raw sequence to the assembled CP genome to verify the assembly results. Then, the reference genome was used to correct the starting position of the CP assembly sequence and determine the position and direction of 4 CP regions (LSC, IRa, SSC, and IRb). The assembly results were imported into Geneiousv.9.0.2 (Kearse et al., 2012) for annotation, and annotations were manually refined using the Geneiousv.9.0.2 (Kearse et al., 2012). Then, the CP genome map was drawn using the online website (https://chlorobox.mpimp-golm.mpg.de/OGDraw.html) (Lohse et al., 2013). At the default Settings, tRNAscanSE (Lowe and Chan, 2016) and DOGMA (Wyman et al., 2004) were used to further validate tRNA genes. Finally, we obtained the gb. File and submit our report to the NCBI. Whole CP genome sequences of *T. obtectum* and *T. serrulatum* were deposited into GenBank with entry numbers PV454981, PV454982, respectively.

### 2.3 Sequence analysis and repeat sequence analysis

Use CodonW software (University of Texas, Houston, TX, United States) to analyze codon usage and relative synonymous codon usage (RSCU), calculate RSCU values, and visualize (Sharp and Li, 1987). Use MEGA X to analyze guanine-cytosine (GC) content (Kumar et al., 2018). The MISA-web (http://pgrc.ipk-gatersleben.de/misa/) was used to identify Simple Sequence Repeats (SSRs) in the *T. obtectum* and *T. serrulatum* CP genomes, with the parameters set to encompass mononucleotide SSRs with 10 repeat units, di- and tri-nucleotide SSRs with 5 and 4 repeat units, respectively, and tetra-, penta-, and hexa-nucleotide SSRs with 3 repeat units. The Long repeat sequences of *T. obtectum* and *T. serrulatum* were detected by REPuter, including forward, palindromic, reverse, and complementary repeats (Kurtz et al., 2001).

### 2.4 Genome comparison and sequence divergence analyses

The mVISTA (v2.0) program in Shuffle-LAGAN mode was used for *Tetrastigma* species CP genome comparison analysis (Frazer et al., 2004). The whole CP genomes were initially aligned using the MAFFT software (Katoh et al., 2019). Then, we used DnaSP 6 (Rozas et al., 2017) software to calculate the nucleotide variability (Pi) with a sliding window of 600 bp and a step size of 200 bp.200 bp step size and a 600 bp window length and the results were visualized using Excel. IRScope software was used to detect the contraction and expansion of the IR/SC region boundaries (Amiryousefi et al., 2018).

### 2.5 ITS2 barcode and secondary structure identification

We collected fresh leaf samples from *T. obtectum* (17 samples) and *T. serrulatum* (20 samples) in Yunnan and identified the two species using conventional barcode ITS2 amplification sequencing. The sample number and location information are listed in Supplementary Table S1. Construct the secondary structures of the ITS2 base sequences of *T. obtectum* and *T. serrulatum* obtained from sequencing using the online tool available at https://www.vectorbuilder.cn/tool/dna-secondary-structure.html. Analyze the differences in their secondary structures by comparing the remaining parameters using an energy minimization-based dynamic programming algorithm.

### 2.6 Phylogenetic analysis

To investigate the phylogenetic relationships within the genus *Tetrastigma* and its placement within the family Vitaceae, we constructed a Maximum Likelihood (ML) phylogenetic tree using CP genome sequences of 46 species from seven genera within the Vitaceae family (*Tetrastigma*, *Parthenocissus*, *Vitis*, *Ampelopsis*, *Ampelocissus*, *Cissus*, and *Leea*), with *Celastrus vaniotii* and *Tripterygium wilfordii* serving as outgroups. The whole CP genomes were initially aligned using the MAFFT software (Katoh et al., 2019). The aligned sequences were then imported into MEGA11 software (Tamura et al., 2021) to determine the best DNA model maximum likelihood (ML). And the best-fit substitution models were selected by ModelTest-NG (Darriba et al., 2020) for ML trees.

## 3 Results and discussion

### 3.1 Chloroplast genome features of *Tetrastigma* species

**FIGURE 1:**
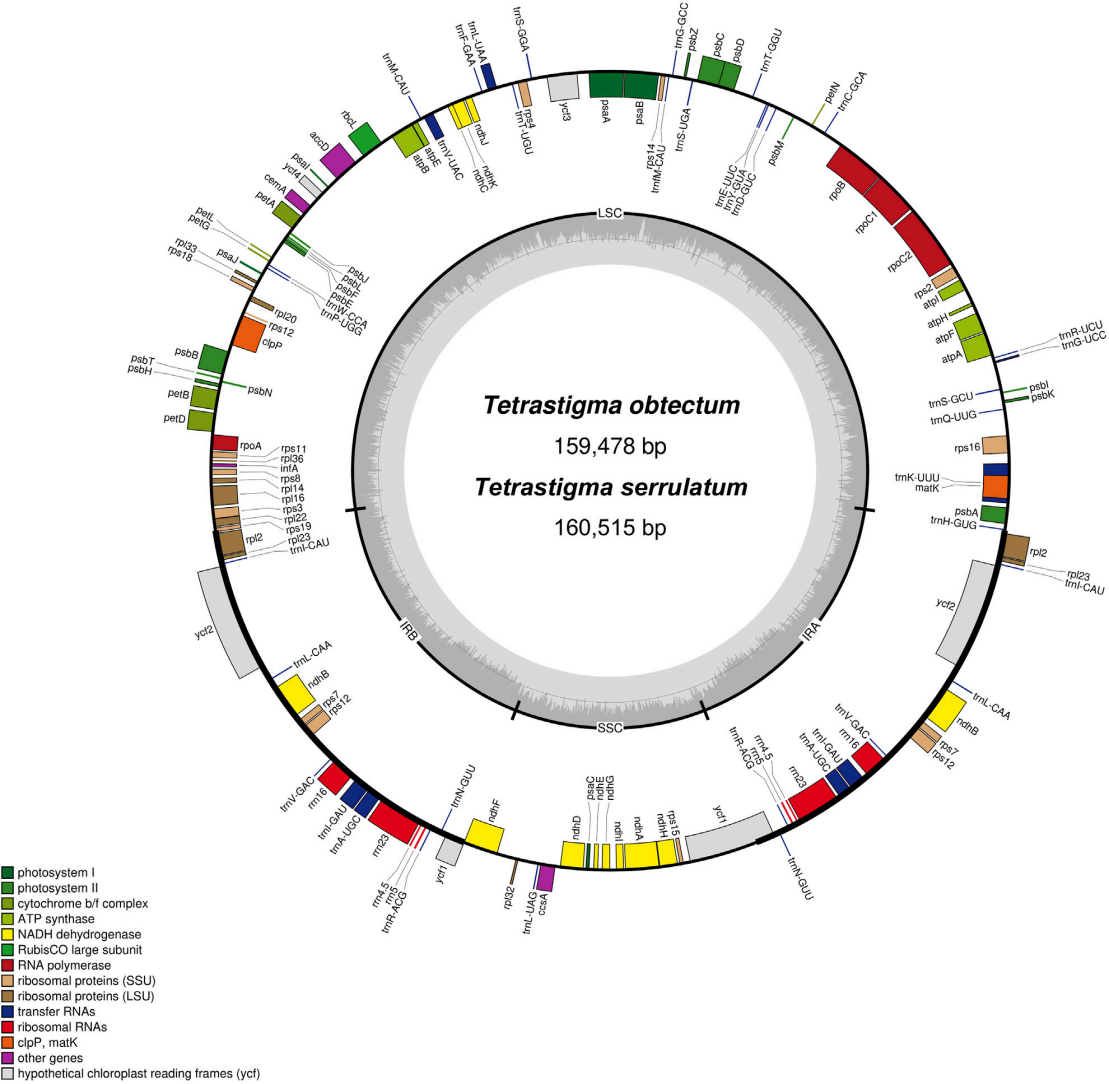
Gene map of two *Tetrastigma* complete chloroplast genomes. Genes on the inside of the outer circle are transcribed in a clockwise direction, while genes on the outside of the outer circle are transcribed in a counterclockwise direction. Genes belonging to different functional categories are different color-coded. The inner circle indicates the range of the LSC, SSC, and IRs. Also, the darker gray area in the inner circle corresponds to the GC content, whereas the lighter gray area corresponds to the AT content.

We organized and analyzed the CP genome characteristics of *T. obtectum* and *T. serrulatum*. The whole genome sequence lengths of *T. obtectum* and *T. serrulatum* CP are 159,478 and 160,515 bp, respectively, both of which have a typical quadripartite structure (Figure 1). The complete CP genomes of *T. obtectum* and *T. serrulatum* consist of a pair of inverted repeats (IRa and IRb) measuring 26,526 bp and 26,480 bp, respectively, an SSC region of 19,045 bp and 19,137 bp, and an LSC region of 87,381 bp and 88,418 bp. The CP genome of *T. serrulatum* is 1,037 bp longer than that of *T. obtectum*. In the whole CP genomes of two *Tetrastigma* species, a total of 131 genes were detected, including 86 different protein-coding genes, 8 different rRNA genes, and 37 different tRNA genes. The gene distribution of the two CP genomes is identical: the LSC region has a total of 81 genes, including 60 protein-coding genes and 21 tRNA genes, while the SSC region contains 11 protein-coding genes and 1 tRNA gene; the IR region replicates a total of 20 genes, including 8 protein-coding genes, 8 tRNA genes, and 4 rRNA genes (Figure 1; Supplementary Table S2).

Additionally, the CP genomes of 14 other *Tetrastigma* species were downloaded from NCBI, allowing for the analysis of 16 species. All CP genomes feature a characteristic quadripartite structure comprising a large single-copy region (LSC), two inverted repeats (IRs), and a small single-copy region (SSC). The chloroplast (CP) genome sizes of the 16 *Tetrastigma* species (Table 2) range from 159,317 base pairs (bp) in *T. pachyphyllum* to 160,893 bp in *T. pyriforme*. The total GC content varies from 37.35% in *T. voinierianum* to 37.62% in *T. cauliflorum*. The length of the LSC region ranges from 87,381 bp in *T. obtectum* to 88,979 bp in *T. pyriforme*. The IR region varies from 26,288 bp in *T. annamense* to 26,934 bp in *T. thorsborneorum*, while the SSC region ranges from 18,649 bp in *T. thorsborneorum* to 19,339 bp in *T. annamense*. The corresponding GC content is 35.34%–35.63%, 42.73%–42.99%, and 31.41%–31.79%, respectively.

**TABLE 2:** Summary of 16 *Tetrastigma* chloroplast genome characteristics.

Name	Genome size	LSC length	SSC length	IR length	Total GC content	GC content of LSC	GC content of IR	GC content of SSC	Number of total genes	Number of CDS	Number of rRNA genes	Number of tRNA genes
*T. serrulatum*	160,515	88,418	19,137	26,480	37.40%	35.50%	42.90%	31.50%	131	86	8	37
*T. obtectum*	159,478	87,381	19,045	26,526	37.50%	35.60%	42.80%	31.50%	131	86	8	37
*T. angustifolium*	159,988	88,107	18,861	26,510	37.55%	35.54%	42.89%	31.77%	131	86	8	37
*T. annamense*	160,736	88,821	19,339	26,288	37.56%	35.35%	42.98%	31.55%	132	87	8	37
*T. canarense*	160,312	88,010	19,048	26,627	37.57%	35.53%	42.82%	31.77%	132	87	8	37
*T. cauliflorum*	160,382	88,238	19,118	26,513	37.62%	35.50%	42.87%	31.65%	131	86	8	37
*T. hemsleyanum*	159,889	87,901	18,966	26,511	37.53%	35.58%	42.89%	31.77%	132	87	8	37
*T. lawsonii*	160,360	88,117	19,306	26,469	37.53%	35.50%	42.93%	31.57%	131	86	8	37
*T. leucostaphylum*	159,387	87,499	18,876	26,506	37.43%	35.59%	42.88%	31.71%	131	86	8	37
*T. nilagiricum*	160,116	88,021	19,111	26,492	37.58%	35.63%	42.90%	31.69%	132	87	8	37
*T. pachyphyllum*	159,317	87,384	18,875	26,529	37.49%	35.62%	42.86%	31.79%	131	86	8	37
*T. planicaule*	160,323	88,181	19,096	26,523	37.39%	35.52%	42.87%	31.66%	131	86	8	37
*T. pyriforme*	160,893	88,979	19,276	26,319	37.48%	35.35%	42.97%	31.51%	131	86	8	37
*T. rafflesiae*	159,805	87,920	19,273	26,306	37.55%	35.63%	42.99%	31.62%	131	86	8	37
*T. thorsborneorum*	160,676	88,159	18,649	26,934	37.48%	35.43%	42.73%	31.62%	131	86	8	37
*T.voinierianum*	160,005	87,874	19,111	26,510	37.35%	35.59%	42.87%	31.65%	131	86	8	37

### 3.2 Codon usage

**FIGURE 2:**
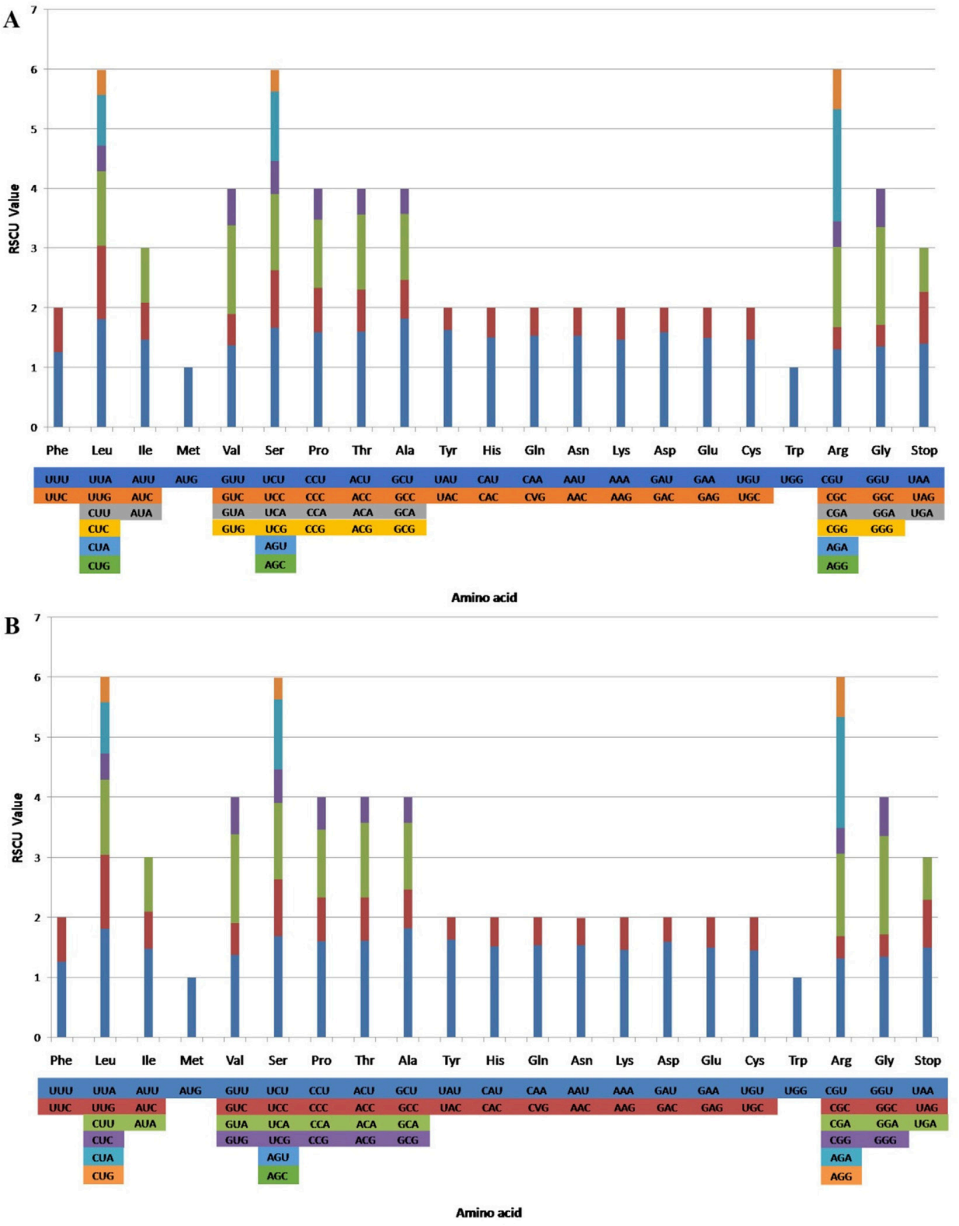
Codon contents in all protein-coding genes in the *T. obtectum* **(A)** and *T. serrulatum* **(B)** complete chloroplast (CP) genome. RSCU, relative synonymous codon usage.

RSCU ratio is an indicator used to measure whether synonymous and non-synonymous codons are used uniformly in a coding sequence. When the RSCU ratio is < 1.00, the codon usage frequency is lower than expected; when the RSCU ratio is > 1.00, the codon usage frequency is higher than expected (Sharp and Li, 1987; Liu et al., 2018). The codon usage levels of the CP genomes of *T. obtectum* and *T. serrulatum* are shown in (Figure 2; Supplementary Table S3). All protein-coding genes comprise 26,909 and 26,857 codons from the two CP genomes. Among them, leucine (Leu) is the most frequent, with an occurrence frequency of 10.39% and 10.41%, followed by isoleucine (Ile), with an occurrence frequency of 8.56% and 8.53%, and cysteine (Cys) is the least frequent, with an occurrence frequency of 1.19% and 1.20%. In the CP genomes of two *Tetrastigma* species, usage of the codons AUG and UGG-encoding methionine and tryptophan, respectively is not biased (RSCU ratio = 1.00). Most protein-coding genes in the CP genome of land plants use the standard AUG initiator, and the AUG codon shows no bias in the CP genomes of *T. obtectum* and *T. serrulatum* (RSCU = 1). Codons ending in A and/or U account for 69.90% of all protein-coding genes in the CP genomes of *T. obtectum* and *T. serrulatum*. In both CP genomes, codons ending in A and/or T (U) generally have higher RSCU ratios, such as GCU (1.82) encoding alanine and UUA (1.81) encoding leucine. The codon usage pattern can be determined by whether there is a high proportion of A/T component bias. In the CP genomes of higher terrestrial plants, preferred codons are often very high, and the preference for A/T is widespread (Kim and Lee, 2004; Zhang et al., 2021a). Our results also showed that except for Leu-UUG, all types of preferred synonymous codons (RSCU ratio >1.00) in the two *Tetrastigma* species end with A or U. A high RSCU ratio may be attributed to the function of amino acids or the peptide structure needed to avoid transcription errors (Gao et al., 2019; Zhang et al., 2021b). This phenomenon suggests that stable CP genome evolution helps protect CP genes from harmful mutations while improving adaptability to selective pressure (Wang et al., 2016; Ivanova et al., 2017).

### 3.3 Simple sequence repeats analyses

**FIGURE 3:**
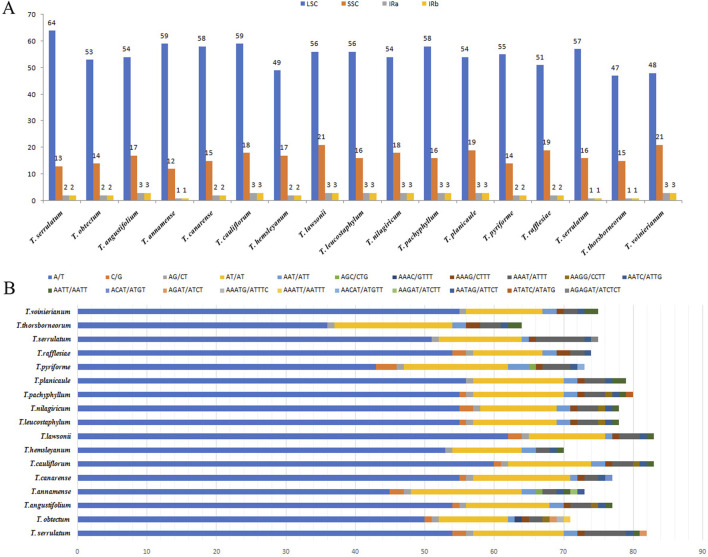
Analysis of chloroplast genome SSRs in 16 *Tetrastigma* species. **(A)** Frequencies of identified SSRs in the large single copy (LSC), SSC and inverted repeats (IRs) regions; **(B)** Frequency of identified SSRs in different repeat class types. The repetitive types of *T. obtectum* and *T. serrulatum* include: mononucleotides, dinucleotides, trinucleotides, tetranucleotides, and pentanucleotides.

Simple sequence repeats are sequences composed of 1-6 nucleotide repeat units, which are widely distributed in CP genomes and are commonly used in population genetics and molecular phylogenetic studies (George et al., 2015; Zhou J. et al., 2018). We analyzed the distribution and types of SSRs in the CP genome by combining 14 species of the genus *Tetrastigma* published by NCBI. A total of 1,217 SSRs were detected. Among them, *T. lawsonii* and *T. cauliflorum* have the highest number of SSRs, 83, while *T. thorsborneorum* has the lowest number of SSRs, with only 64. The SSRs in the sixteen species are primarily distributed in the LSC region of the CP genome, followed by the SSC region and the IR region (Table 3). Mononucleotide repeats (56.25∼77.11%) are the most abundant type, followed by dinucleotides (14.46%–28.13%); tetranucleotide (5.48%–12.5%), trinucleotide (1.2%–5.48%), and pentanucleotide repeat sequences (0%–2.82%). Regarding nucleotide composition types, a total of twenty-one repeat types were detected, mainly A/T and AT/AT (Figure 3). A/T repeat sequences (46.36∼57.63%) are the most abundant, followed by AT/TA dinucleotide repeat sequences (20.34∼28.68%) and AAAT/ATTT tetranucleotide repeat sequences (5.93∼8.18%). A detailed analysis of different nucleotide composition types reveals that A and T are the most commonly used bases, while repeat sequences containing C and G bases are infrequent. Our results are consistent with previous studies reporting that CP SSRs usually consist of short poly-A or poly-T repeats (Kuang et al., 2011; Zhang et al., 2021a). Interestingly, among the 16 species of the genus *Tetrastigma*, only *T. obtectum*, *T. annamense*, *T. pachyphyllum*, and *T. pyriforme* have five nucleotide SSRs. In contrast, no species of the genus *Tetrastigma* has six-nucleotide SSRs. Currently, SSR markers are widely used in fields such as genetic diversity and population structure assessment, comparative genomics, genetic map development, and marker-assisted selection breeding (Flannery et al., 2006; Cui et al., 2019). The repeat sequences identified in this study are a valuable resource for species identification and research on the genetic diversity and population structure of *Tetrastigma* plants.

**TABLE 3:** SSRs characteristics and distribution of chloroplast genomes in 16 *Tetrastigma* species.

Species	Mono	Di	Tri	Tetra	Penta	Hexa	Total	LSC	SSC	Ira	Irb
*T. serrulatum*	56	14	2	10	0	0	82	64	13	2	2
*T. obtectum*	51	11	1	6	2	0	71	53	14	2	2
*T. angustifolium*	55	13	2	7	0	0	77	54	17	3	3
*T. annamense*	47	17	3	4	2	0	73	59	12	1	1
*T. canarense*	56	15	1	5	0	0	77	58	15	2	2
*T. cauliflorum*	61	13	2	7	0	0	83	59	18	3	3
*T. hemsleyanum*	53	11	2	4	0	0	70	49	17	2	2
*T. lawsonii*	64	12	1	6	0	0	83	56	21	3	3
*T. leucostaphylum*	56	13	2	7	0	0	78	56	16	3	3
*T. nilagiricum*	57	12	2	7	0	0	78	54	18	3	3
*T. pachyphyllum*	56	14	2	7	1	0	80	58	16	3	3
*T. planicaule*	56	14	2	7	0	0	79	54	19	3	3
*T. planicaule*	46	16	4	6	1	0	73	55	14	2	2
*T. rafflesiae*	56	11	2	5	0	0	74	51	19	2	2
*T. thorsborneorum*	36	18	2	8	0	0	64	47	15	1	1
*T. voinierianum*	55	12	2	6	0	0	75	48	21	3	3

### 3.4 Contraction and expansion of IRs

**FIGURE 4:**
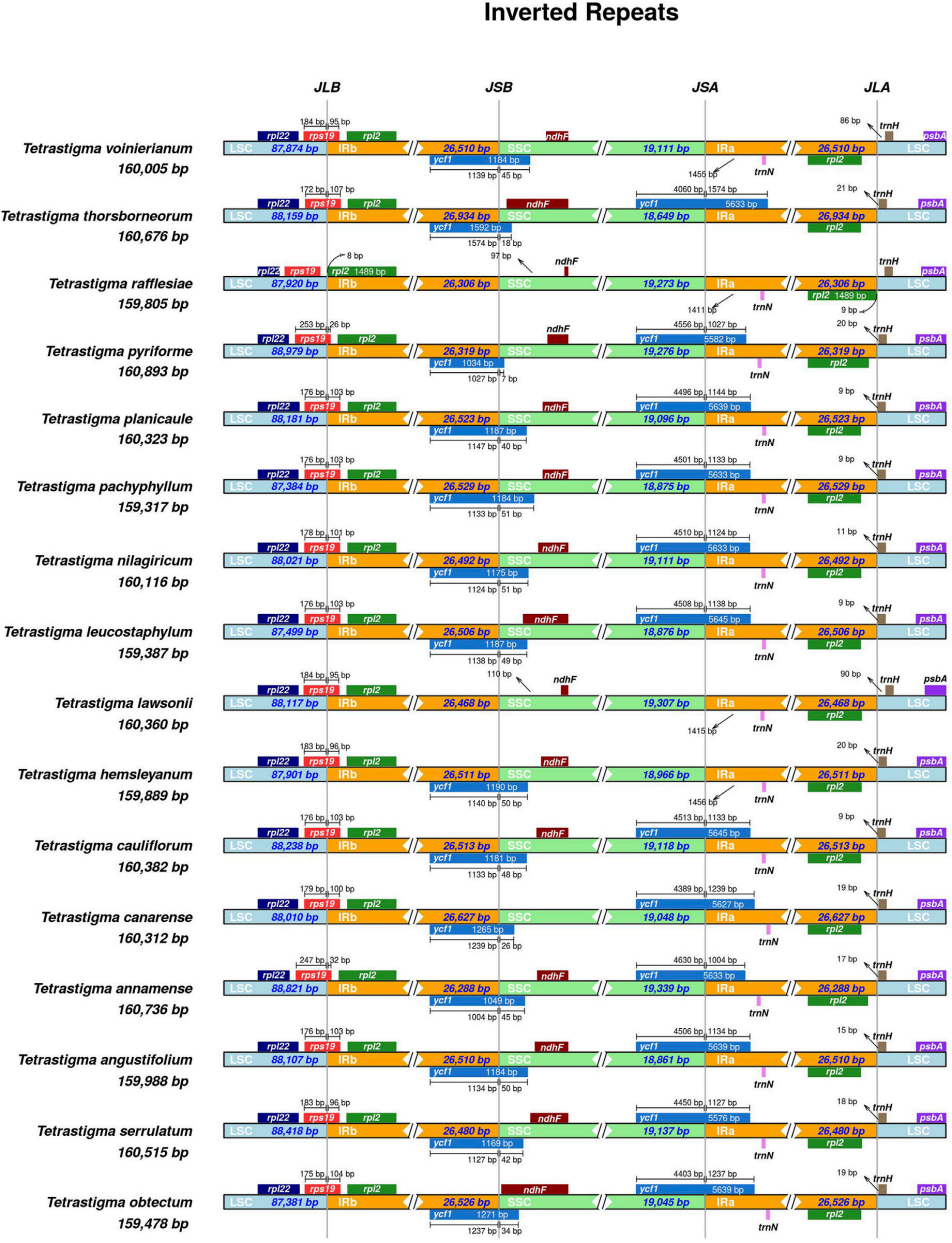
Comparison of the borders of the LSC, SSC, IRs regions among four CP genomes of *Tetrastigma*. JLB: boundary of the LSC and the IRb. JSB: boundary of the SSC and the IRb. JSA: boundary of the SSC and the IRa. JLA: boundary of the LSC and the IRa.

The CP genome of higher plants is highly conserved. Many researchers believe that the main reason for the variation in CP genome size is the contraction and expansion of the IR regions and the boundaries of the LSC/SSC regions (Wang et al., 2022; Zhang et al., 2024). In this study, we compared the IR/LSC and IR/SSC boundary structures of 16 species of *Tetrastigma*. The expansion and contraction of the IRs are shown in (Figure 4). The results indicate that the length of the IR region in the 16 species of ivy ranges from 26,306 to 26,934 bp, with no significant differences. The *psb*A gene positions of all species in the genus *Tetrastigma* are similar and are completely located in the LSC region. The *trn*H gene is also located within the LSC region, with a distance to the boundary ranging from 9 to 90 bp. The *rps*19 gene in *T. rafflesiae* is located in the LSC region, while in the other 15 species of the genus *Tetrastigma*, it spans the LSC/IRb boundary, with the portion within IRb ranging from 26 to 107 bp. The *ndh*F genes of 16 species of the genus *Tetrastigma* are all located in the SSC region, with distances from the IRb/SSC boundary ranging from 5 to 110 bp. The *ycf*1 gene (5,576–5,645 bp) spans the SSC/IRa boundary, and a portion of the *ycf*1 gene (1,034–1,265) spans the IRb/SSC region were annotated as pseudogenes. This also leads to the *ycf*1 pseudogene and the gene *ndh*F having partial overlap in the SSC region in the three species *T. thorsborneorum*, *T. leucostaphylum*, and *T. obtectum*. The pseudogenization and copy positions of *ycf*1 are also frequently observed in other higher plants (Lu et al., 2022; Zhu et al., 2024). In summary, while the CP genomes of the 16 *Tetrastigma* species exhibit high conservation in gene number and genomic structure, there are notable differences in the lengths of specific genes and the IR, LSC, and SSC regions. This phenomenon suggests that the expansion and contraction of the IR region may primarily drive changes in CP genome length and serve as a catalyst for variations in plant CP genomes (Kim and Lee, 2004; Pei et al., 2022; Zhang et al., 2024).

### 3.5 Comparative chloroplast genome analysis

**FIGURE 5:**
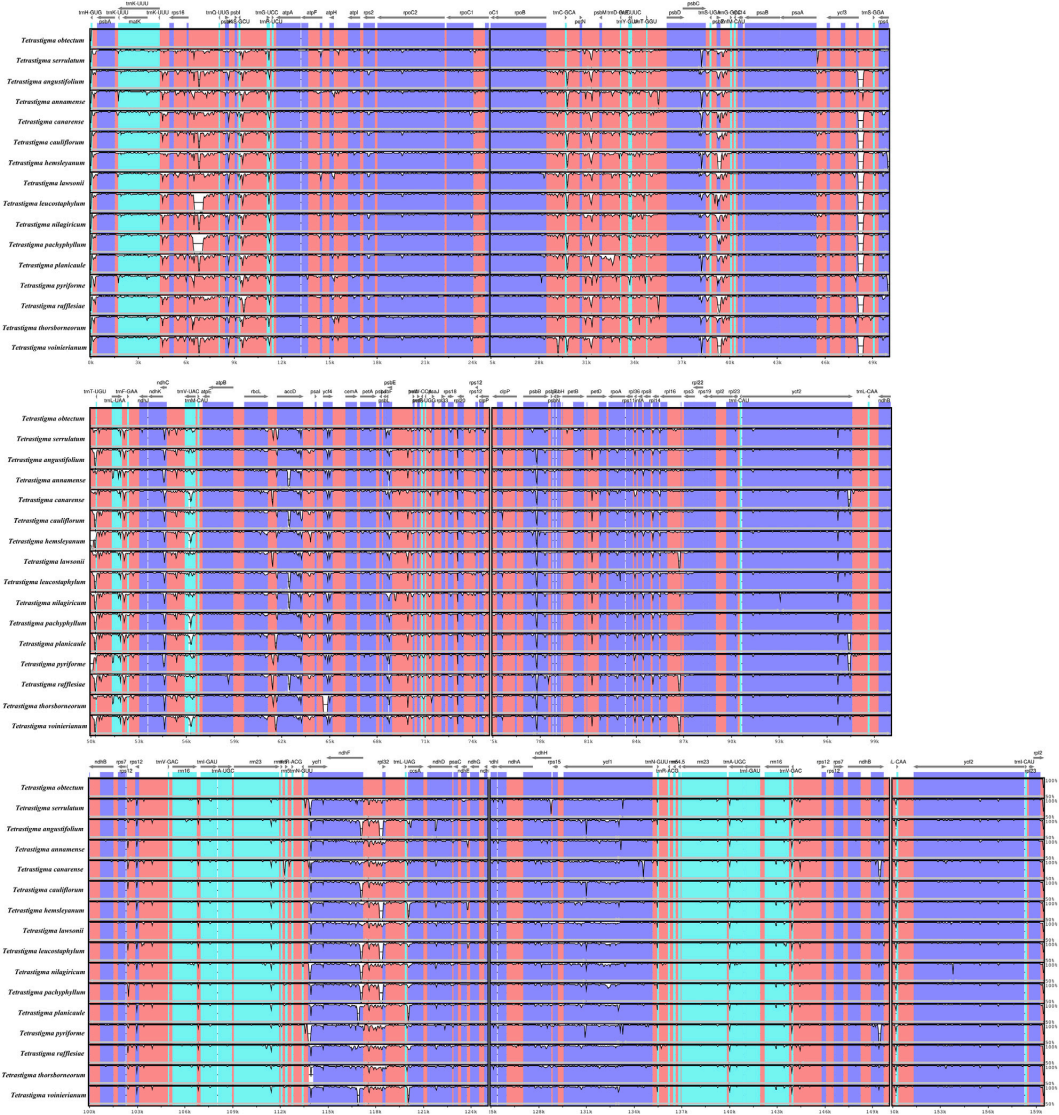
The alignment and comparative analysis of the whole CP genome for sixteen *Tetrastigma* species using mVISTA, and *T. obtectum* as a reference. Gray arrows and thick black lines above the alignments indicate gene orientations. White peaks represent differences among CP genomes. Exons, introns, and conserved noncoding sequences (CNSs) were displayed in different colors. A similarity cut-off value of 70% was used for the plots, and the Y-axis represents the percentage similarity (50%–100%).

The genomic structure of plant CP is highly conserved, and through comparative analysis, high mutation regions can be easily identified. These variations contribute to elucidating the genetic structure and evolutionary relationships of this genus of plants (Daniell et al., 2016; Hong et al., 2020). This study conducted a comparative analysis of a total of 16 *Tetrastigma* CP genomes, and the results showed the overall differences among the 16 CP genomes are minimal, with the sequence differences in the two IR regions being smaller than those in the LSC and SSC regions (Figure 5). Additionally, we found that the sequence differences are more prominent in non-coding regions, whereas the differences in exons and untranslated regions (UTRs) are more minor. The most differentiated non-coding regions include *rps*16-*trn*G-UUC,*psb*M-*trn*D-GUC, *psb*E-*pet*L, *ndh*F-*rpl*32-*trn*L-UAG, and the most differentiated coding regions include *rpl*16, *rpl*22,*ycf*1. This result further supports the view held by many scholars that the coding regions of higher plants are more conserved than non-coding regions, and the IR regions are more conserved than the LSC and SSC regions (Nazareno et al., 2015; Fan and Ma, 2022; Zhang et al., 2024). In the study Elimination of deleterious mutations in plastid genomes by gene conversion, Khakhlova believes that due to gene conversion correcting mutations in the IR sequence (Khakhlova and Bock, 2006).

### 3.6 Discovery of candidate markers based on nucleotide diversity analyses

**FIGURE 6:**
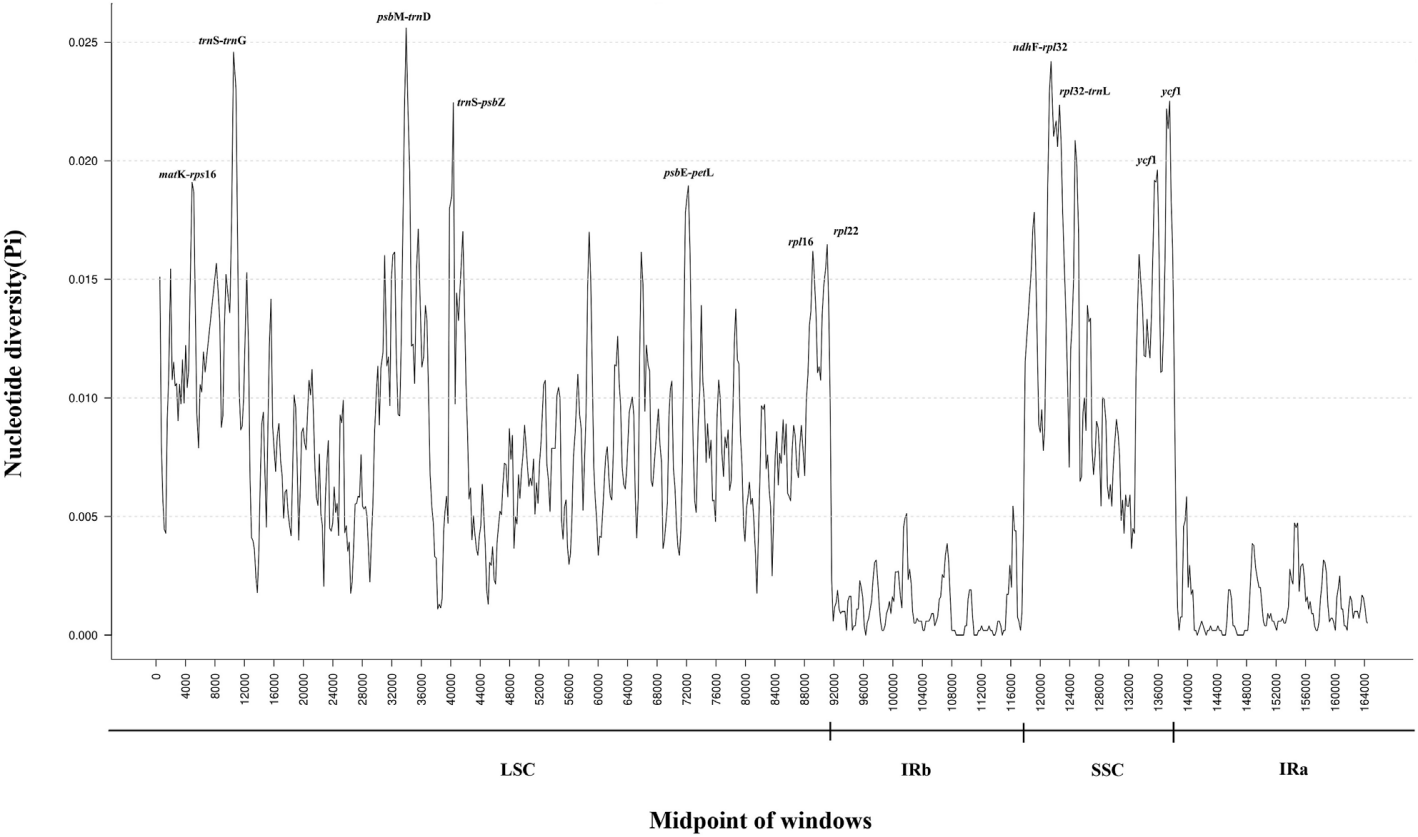
Sliding window analysis based on the complete chloroplast (CP) genomes of sixteen *Tetrastigma* species. Window length: 600 bp; step size: 200 bp. X-axis: position of the midpoint of a window. Y-axis: nucleotide diversity of each window.

Nucleotide polymorphism (Pi) analysis of CP genomes from 16 *Tetrastigma* species was conducted using DnaSP software, identifying highly variable regions (Figure 6). The IR region exhibits lower variability than the LSC and SSC regions, similar to previous studies. The results showed that the average Pi value of 16 species of the genus *Tetrastigma* was 0.0065 (Supplementary Table S4). A total of 6 nucleotide polymorphism hotspots with Pi > 0.02 were detected, with 3 found in the LSC region: *trn*S-GCU-*trn*G-UCC, *psb*M-*trn*D-GUC, *trn*S-UGA-*psb*Z, and 3 found in the SSC region: *ndh*F-*rpl*32, *rpl*32-*trn*L-UAG, *ycf*1. These results suggest that the LSC and SSC regions of *Tetrastigma* species may have experienced rapid nucleotide substitution, which is crucial for species identification and phylogenetic analysis. This study performed a nucleotide polymorphism (Pi) analysis on the CP genomes of 16 *Tetrastigma* species. The ten regions with the highest variability detected are *psb*M-*trn*D-GUC, *ndh*F-*rpl*32, *trn*S-GCU-*trn*G-UCC, *ycf*1, *rpl*32-*trn*L-UAG, *trn*S-UGA-*psb*Z, *psb*E-*pet*L, *mat*K-*rps*16, *rpl*16, and *rpl*22. These highly variable regions can be candidate markers for species identification within the genus *Tetrastigma*. Previous molecular identification studies on *Panax*, *Dracaena*, and *Gentiana* have demonstrated that CP genetic markers possess high identification capability (Nguyen et al., 2017; Zhou T. et al., 2018).

### 3.7 ITS2 barcode and secondary structure identification

**FIGURE 7:**
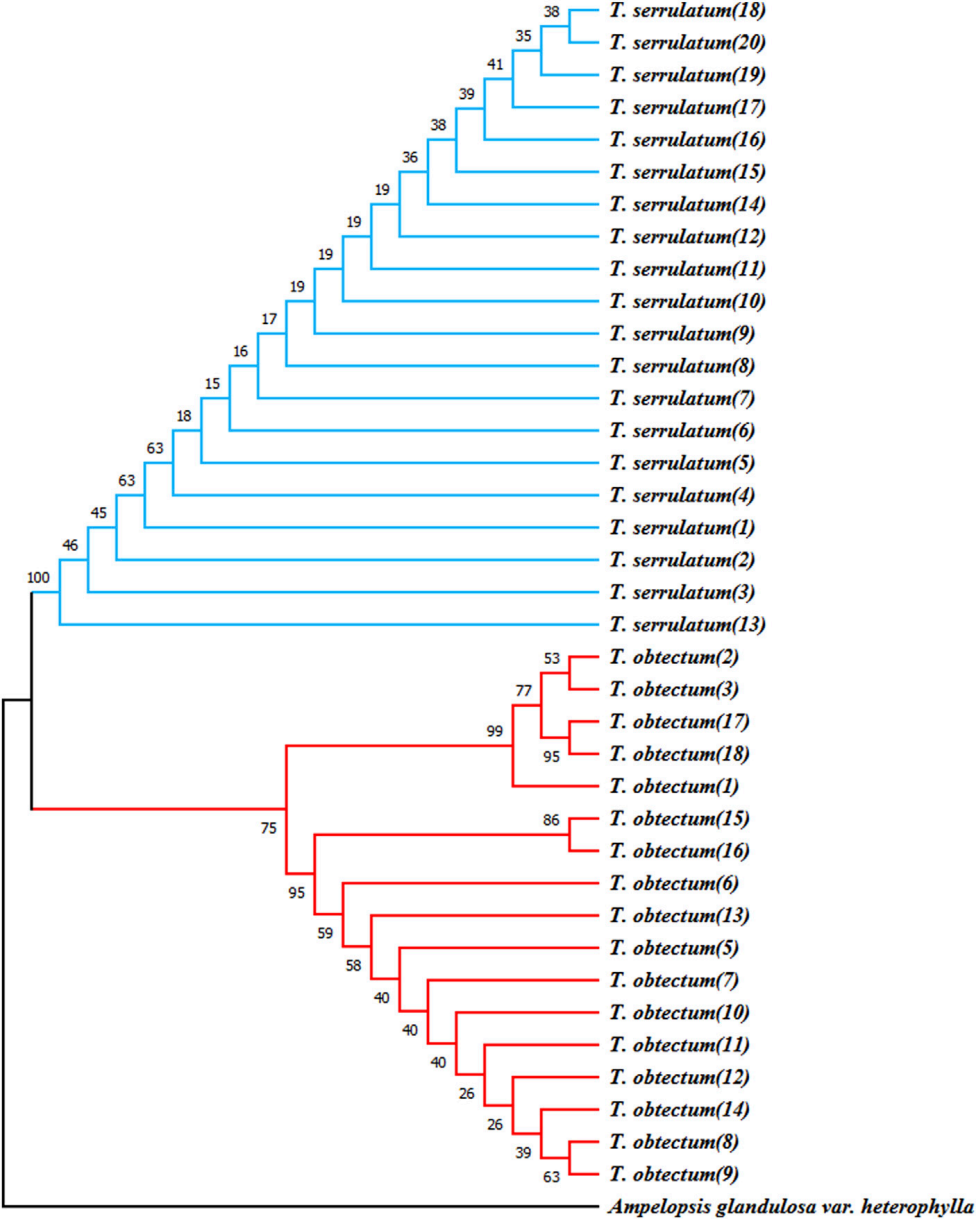
Neighbor-joining bootstrap trees (based on Kimura-2-Parameter) illustrating the resolution of *T. obtectum* and *T. serrulatum* for the “ITS2” barcoding.

**FIGURE 8:**
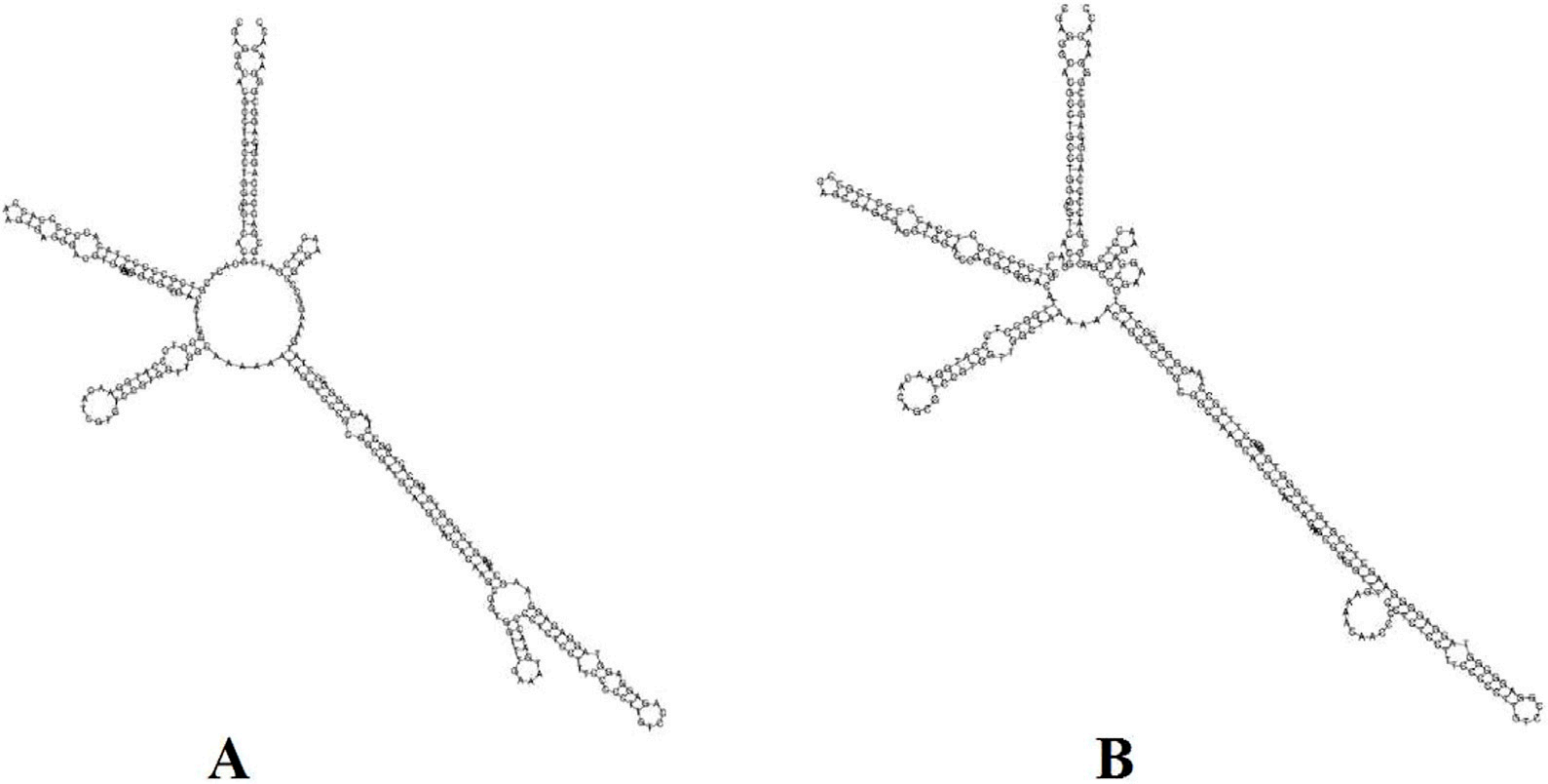
Constructing secondary structures *T. serrulatum* and *T. obtectum* based on ITS2 base sequence, **(A)** is the secondary structure of *T. serrulatum*, while the secondary structure of *T. obtectum* includes both **(A, B)**.

To identify the most effective barcode markers for species within the genus *Tetrastigma*, we utilized the conventional DNA barcode ITS2 to amplify two *Tetrastigma* species and evaluated their identification efficiency. We constructed an NJ phylogenetic tree using conventional barcodes (Figure 7). The results show that variable sites in the ITS2 base sequence can effectively distinguish *T. obtectum* from *T. serrulatum*. Additionally, we constructed the secondary structures of the ITS2 sequences for *T. obtectum* and *T. serrulatum* using an online tool, as depicted in Figure 8. According to the secondary structure diagrams of the ITS2 base sequences, *T. serrulatum* exhibits a unique secondary structure (Figure 8A), whereas *T. obtectum* displays two distinct secondary structures (Figures 8A,B). Notably, the structure in Figure 8A shares the same stem and loop configuration as that of *T. serrulatum*. Consequently, it is not feasible to differentiate these two species based on the shape of the ITS2 secondary structures alone. In this study, the traditional barcode ITS2 base sequence successfully distinguished *T. obtectum* from *T. serrulatum*. However, the limited number of sampled species in this study necessitates an expansion of sampling in future research. It is recommended that future studies increase the sampling scope within the genus and incorporate CP barcode markers for more comprehensive species identification.

### 3.8 Phylogenetic relationships of Vitaceae species

**FIGURE 9:**
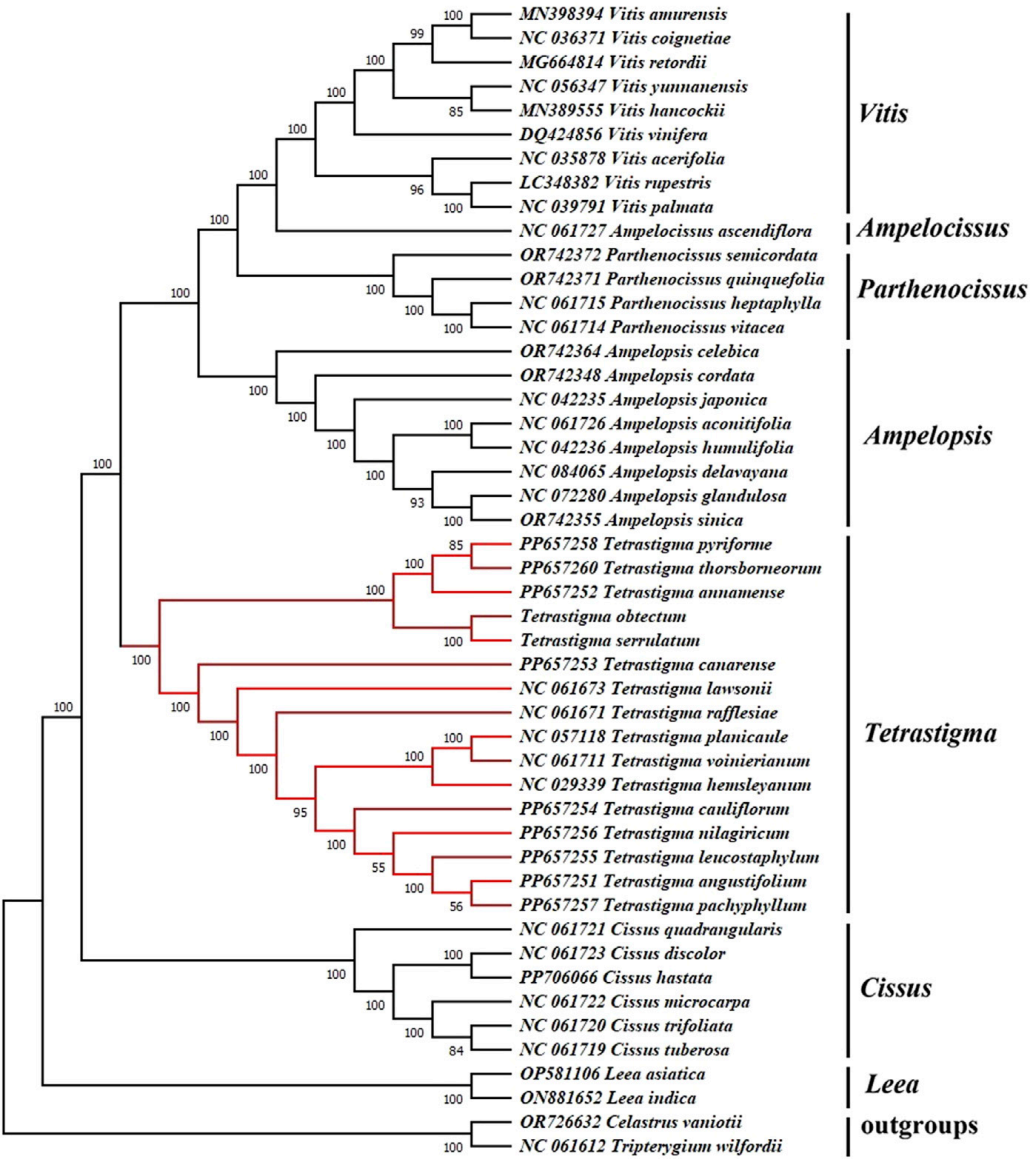
Maximum Likelihood (ML) phylogenetic tree based on complete chloroplast genomes, with *Celastrus vaniotii* and *Tripterygium wilfordii* as outgroups. Bootstrap values are displayed on the branch phylogenetic tree, the number above each node referred to the bootstrap value from 1,000 replicates and the optimal model selected by ModelTest-NG is “GTR + I + G”.

The CP genome sequence is crucial for investigating the phylogenetic relationships, genetic structure, and taxonomic classification of higher plants. To investigate the phylogenetic relationships within the genus *Tetrastigma* and its placement within the family Vitaceae, we constructed a Maximum Likelihood (ML) phylogenetic tree using CP genome sequences of 46 species from Vitaceae family (Figure 9). And all nodes received strong support. The phylogenetic tree indicates that the *L. asiatica* and *L. indica* from the genus *Leea* are the first to split into a separate branch. At the same time, the remaining species are grouped into another significant branch. Then, the six species of the *Cissus* divided into one branch, while the species of the remaining five genera split into a large branch. Next, the species of the genus *Tetrastigma* gathered on one branch, and each species was separated. In contrast, the species of *Parthenocissus*, *Vitis*, *Ampelopsis*, and *Ampelocissus* gathered on another branch, with a support rate of 100%. In *Tetrastigma*, *T. obtectum*, *T. serrulatum*, *T. annamense*, *T. thorsborneorum*, and *T. pyriforme* form one clade, while the remaining species form another clade, both supported by 100% bootstrap values. This result aligns with the conclusion of Zhu et al. (2024) that species of the genus *Tetrastigma* constitute a monophyletic group. In the classification study by Zhu et al. (2024), the close phylogenetic relationship between *T. thorsborneorum* and *T. pyriforme* has been confirmed. Additionally, the phylogenetic analysis of the species from the Vitaceae family included in this study is more comprehensive, resulting in a more stable and accurate phylogenetic relationship. Therefore, we believe the CP genome can be a reliable tool for species identification within the genus *Tetrastigma*. As a highly effective super barcode, the CP genome sequence offers a robust method for identifying higher plant species. Furthermore, we believe that as more species are included in the analysis, the species relationships within this genus will change and become clearer. The genus *Tetrastigma* includes numerous species, many of which have uncertain classification statuses and *Tetrastigma* relationships. Future phylogenetic analyses should incorporate more CP genome samples. Our results provide valuable references and a foundation for species identification using the CP genome to reveal the evolutionary relationships within this genus and help to improve the understanding of the phylogeny of the *Tetrastigma* genus.

## 4 Conclusion

This study assembled and annotated the complete CP genomes of *T. obtectum* (159,478 bp) and *T. serrulatum* (160,515 bp) and analyzed them in combination with the existing CP genomes of 14 *Tetrastigma* species from NCBI. The CP genomes of *Tetrastigma* exhibited a typical circular tetramerous structure, including a large single-copy region (87,381–88,979 bp), a small single-copy region (18,649–19,339 bp), and a pair of inverted repeats (26,288–26,934 bp). The guanine-cytosine content of the CP genomes is 37.35%–37.62%. The codon usage of *Tetrastigma* plants shows a significant preference for ending with A/T. A total of 1,217 SSRs were detected in 16 species of *Tetrastigma*. We then compared the analysis of the CP genome sequences of *Tetrastigma* species and analyzed their sequence characteristics. Nucleotide diversity was analyzed based on the alignment of complete CP genome sequences, identifying ten polymorphic hotspots suitable for DNA marker development. Using the ITS2 sequence and its secondary structure for species identification of *T. obtectum* and *T. serrulatum*, our results demonstrate that the ITS2 sequence can effectively discriminate between the two species, whereas the secondary structure cannot. Finally, to investigate the phylogenetic relationships within the genus *Tetrastigma* and its placement within the family Vitaceae, we constructed a Maximum Likelihood (ML) phylogenetic tree using CP genome sequences of 46 species from seven genera within the Vitaceae family (*Tetrastigma*, *Parthenocissus*, *Vitis*, *Ampelopsis*, *Ampelocissus*, *Cissus*, and *Leea*).

## Data Availability

The GenBank data presented in the study are deposited in the NCBI (Nucleotide) repository, available at: https://www.ncbi.nlm.nih.gov/nuccore/2987893153 and https://www.ncbi.nlm.nih.gov/nuccore/PV454981. Further inquiries can be directed to the corresponding authors.
