# Supplementary material for: Molecular identification and phylogenetic analysis of confusing Tetrastigma species based on DNA barcoding and chloroplast genome
Source: Front Pharmacol. 2025 Jul 11;16:1607947. doi: 10.3389/fphar.2025.1607947 (PMC12289577; doi:10.3389/fphar.2025.1607947)
Supplement: Supplementary file 3 [file Table1.doc]

| **Species-No.** | **Location** | **acquisition time** |
| --- | --- | --- |
| *Tetrastigma obtectum(1)* | Mangshi,Yunnan Province,China | November, 2021 |
| *Tetrastigma obtectum(2)* | Mangshi,Yunnan Province,China | November, 2021 |
| *Tetrastigma obtectum(3)* | Mangshi,Yunnan Province,China | November, 2021 |
| *Tetrastigma obtectum(5)* | Mangshi,Yunnan Province,China | November, 2021 |
| *Tetrastigma obtectum(6)* | Tengchong,Yunnan Province,China | October, 2021 |
| *Tetrastigma obtectum(7)* | Tengchong,Yunnan Province,China | October, 2021 |
| *Tetrastigma obtectum(8)* | Tengchong,Yunnan Province,China | October, 2021 |
| *Tetrastigma obtectum(9)* | Tengchong,Yunnan Province,China | October, 2021 |
| *Tetrastigma obtectum(10)* | Tengchong,Yunnan Province,China | October, 2021 |
| *Tetrastigma obtectum(11)* | Dali,Yunnan Province,China | October, 2021 |
| *Tetrastigma obtectum(12)* | Dali,Yunnan Province,China | October, 2021 |
| *Tetrastigma obtectum(13)* | Dali,Yunnan Province,China | October, 2021 |
| *Tetrastigma obtectum(14)* | Dali,Yunnan Province,China | October, 2021 |
| *Tetrastigma obtectum(15)* | Dali,Yunnan Province,China | October, 2021 |
| *Tetrastigma obtectum(16)* | Honghe,Yunnan Province,China | November, 2021 |
| *Tetrastigma obtectum(17)* | Honghe,Yunnan Province,China | November, 2021 |
| *Tetrastigma obtectum(18)* | Honghe,Yunnan Province,China | November, 2021 |
| *Tetrastigma serrulatum(1)* | Heqing,Yunnan Province,China | October, 2021 |
| *Tetrastigma serrulatum(2)* | Heqing,Yunnan Province,China | October, 2021 |
| *Tetrastigma serrulatum(3)* | Nanjian,Yunnan Province,China | October, 2021 |
| *Tetrastigma serrulatum(4)* | Yingjiang,Yunnan Province,China | November, 2021 |
| *Tetrastigma serrulatum(5)* | Yingjiang,Yunnan Province,China | November, 2021 |
| *Tetrastigma serrulatum(6)* | Yingjiang,Yunnan Province,China | November, 2021 |
| *Tetrastigma serrulatum(7)* | Yingjiang,Yunnan Province,China | November, 2021 |
| *Tetrastigma serrulatum(8)* | Yingjiang,Yunnan Province,China | November, 2021 |
| *Tetrastigma serrulatum(9)* | Tengchong,Yunnan Province,China | October, 2021 |
| *Tetrastigma serrulatum(10)* | Tengchong,Yunnan Province,China | October, 2021 |
| *Tetrastigma serrulatum(11)* | Tengchong,Yunnan Province,China | October, 2021 |
| *Tetrastigma serrulatum(12)* | Tengchong,Yunnan Province,China | October, 2021 |
| *Tetrastigma serrulatum(13)* | Tengchong,Yunnan Province,China | October, 2021 |
| *Tetrastigma serrulatum(14)* | Dali,Yunnan Province,China | October, 2021 |
| *Tetrastigma serrulatum(15)* | Dali,Yunnan Province,China | October, 2021 |
| *Tetrastigma serrulatum(16)* | Lushui,Yunnan Province,China | August, 2021 |
| *Tetrastigma serrulatum(17)* | Lushui,Yunnan Province,China | August, 2021 |
| *Tetrastigma serrulatum(18)* | Lushui,Yunnan Province,China | August, 2021 |
| *Tetrastigma serrulatum(19)* | Lushui,Yunnan Province,China | August, 2021 |
| *Tetrastigma serrulatum(20)* | Lushui,Yunnan Province,China | August, 2021 |
